# Investigating Strategies to Enhance the Aqueous Solubility of Ketamine HCl for Intranasal Delivery

**DOI:** 10.3390/pharmaceutics16121502

**Published:** 2024-11-22

**Authors:** Sourour Idoudi, Alaaeldin Saleh, Mohammed Akkbik, Leena Amine, Khalid Alansari, Ousama Rachid, Alaaldin M. Alkilany

**Affiliations:** 1 Department of Pharmaceutical Sciences, College of Pharmacy, Qatar University, Doha P.O. Box 2713, Qatar; si1602796@qu.edu.qa (S.I.); m.akkbik@qu.edu.qa (M.A.); orachid@qu.edu.qa (O.R.); 2 College of Medicine, Qatar University, Doha P.O. Box 2713, Qatar; alaaeldin.saleh@qu.edu.qa; 3 Central Laboratories Unit, Office of VP for Research & Graduate Studies, Qatar University, Doha P.O. Box 2713, Qatar; 4 Department of Emergency, Sidra Medicine, Doha P.O. Box 26999, Qatar; lamine@sidra.org (L.A.); kalansari@sidra.org (K.A.)

**Keywords:** ketamine HCl, solubility, sodium dodecyl sulfate, solvent, intranasal delivery

## Abstract

**Background:** Ketamine HCl, an FDA-approved therapeutic, is administered through various routes, including intranasal delivery. Administering an adequate therapeutic dose of intranasal ketamine HCl is challenging due to the limited volume that can be delivered intranasally given the current commercially available concentrations. **Objectives:** This study investigates solubilizing strategies to enhance the aqueous solubility of ketamine HCl for intranasal administration. **Methods:** We assessed the solubility profile of ketamine HCl by evaluating factors such as pH, co-solvents, and surfactants. Additionally, we developed and validated a UV-Vis spectroscopy method for ketamine HCl analysis. **Results:** Our solubility screening in various organic co-solvents revealed the following order of effectiveness in enhancing solubility: methanol > water > propylene glycol > ethanol > dimethyl sulfoxide (DMSO) > N-methyl-2-pyrrolidone (NMP). Despite methanol’s superior solubility, its potential toxicity, coupled with the relatively lower effectiveness of other solvents compared to water, suggests that a co-solvency approach is not advantageous for ketamine HCl. We found that ketamine HCl solubility increased with medium acidity, with pH 3.5 being the optimal for further formulation studies. The impact of pharmaceutical surfactants on ketamine HCl solubility at an acidic pH was also evaluated. Surfactants tested included SDS, PEG 400, PVP, Tween 20, poloxamer 188, and lecithin. Notably, PEG 400 and PVP reduced solubility due to a salting-out effect, whereas Tween 80, lecithin, and poloxamer 188 slightly improved solubility through micelle formation. Among the surfactants tested, 1% SDS emerged as the most effective in enhancing ketamine HCl solubility. **Conclusions:** These outcomes highlight the potential of these solubilization strategies to address the solubility limitations of ketamine HCl, enabling the preparation of highly concentrated ketamine HCl formulations for intranasal delivery.

## 1. Introduction

**Figure 1:**
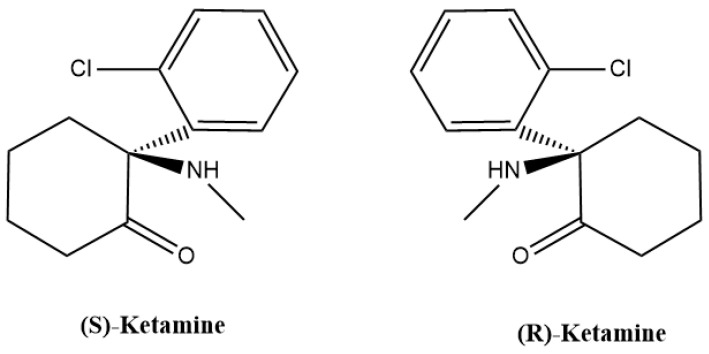
Chemical structure of ketamine enantiomers. Both stereoisomers, (S)-ketamine and (R)-ketamine, are non-superimposable mirror images. Figure created in ChemDraw.

In the 1950s, Parke-Davis industries initiated the search for an ideal anesthetic product with analgesic potential among the derivatives of phencyclidine drugs [1]. Among these trials, the synthesis of ketamine had emerged in the 1960s [2,3]. Ketamine, also named 2-O-chlorophenyl-2-methylamino cyclohexanone, is an approved pharmaceutical by the Food and Drug Administration (FDA) and possesses a chemical formula of C_13_H_16_ClNO [4]. Moreover, ketamine consists of a central chiral carbon, thus enabling two different steric configurations. Furthermore, ketamine is a racemic mixture that consists of equal amounts of two enantiomers, (S)-ketamine and (R)-ketamine (Figure 1) [5]. These two enantiomers have different affinities for different receptors, and consequently somewhat different clinical profiles [5,6,7]. For instance, the FDA approved an intranasal (S)-ketamine formulation (Spravato^®^) as an antidepressant drug in 2019 [8,9,10].

As a pharmaceutical, ketamine possesses versatile biomedical applications including procedural sedation and depression treatment, in addition to off-label uses such as asthma treatment and pain management [9,11,12,13]. Various administration routes, including intravenous (IV), intramuscular, sublingual, oral, and intranasal (IN) have been reported for ketamine [10,14]. Nowadays, IN drug delivery is becoming a potential alternative to other drug administration routes because the nasal cavity is highly supplied with blood vessels that facilitate the rapid absorption of drugs, leading to the enhanced bioavailability of the drug in the site of action in a non-invasive method [15,16]. Thus, the IN route offers a combination of rapid absorption, improved bioavailability, convenience, and the potential for both systemic and localized effects, making it a valuable option for drug delivery. However, variations in the administration route of ketamine might need dose adjustments to achieve the intended therapeutic effect [1,17,18]. For instance, the IN delivery of ketamine is mainly affected by the drug’s solubility, stability, and the pH of the administered dose [19]. Moreover, the nasal cavity has a limited volume capacity, where concentrated doses in a small volume ranging between 0.2 and 0.3 mL per nostril are considered the ideal volumes, whereas larger volumes would not be reliably absorbed due to mucosal surface saturation and runoff from the nasal cavity [20,21,22]. Usually, ketamine doses ranging from 3 to 5 mg/kg are used for mild sedation, and thus, it is necessery to solubilize 200–300 mg/mL of ketamine HCl, which exceeds its solubility limit [23,24]. In addition, in our ongoing interest to evaluate the sedative efficacy of an IN ketamine HCl formulation, we are planning to compare it to the standard IV ketamine HCl administration in patients, which requires a large quantity of ketamine HCl to be dissolved in a unit volume of medium suitable for IN delivery, prompting the investigation presented in this work.

There are several approaches to improving drug solubility and its IN delivery, including pH optimization as well as the incorporation of co-solvents or surfactants [25,26,27]. The choice of the solubilization approach could considerably affect the drug’s solubility, stability, and bioavailability in the site of action [28], as well as prevent its precipitation, degradation, and aggregation during storage [29,30]. The pH is a critical variable controlling the solubility of ionizable drugs, and this is the case with ketamine HCl, which is a weak base drug (pKa of 7.5) [31,32]. Various co-solvents can be used to improve a drug’s solubility, including methanol, ethanol, propylene glycol (PG), dimethyl sulfoxide (DMSO), dimethylformamide (DMF) and others. Pharmaceutical surfactants and solubilizing agents are abundant and range from molecular surfactants and polymeric surfactants to polymers and various types of nanovehicles. In addition, ketamine HCl is preferred for IN formulations due to its higher aqueous solubility, which is essential for its effective delivery through the nasal route. Unlike the free base, ketamine HCl dissolves readily in water, especially at acidic pH levels, making it suitable for the limited volume available for IN administration. It is also important to note that this salt form of ketamine is used in FDA-approved intravenous injections currently available on the market. The literature is abundant with reports detailing the enhancement of the solubility of various drugs using these solubilizing approaches; however, there are no such studies available for ketamine HCl. Furthermore, as ketamine HCl is a widely used therapeutic agent, it is crucial to develop and validate analytical methods for its quantification in pharmaceutical dosage forms. The literature reports methods such as high-performance liquid chromatography (HPLC), mass spectrometry (MS), and gas chromatography (GC), all of which require trained personnel, and significant time, cost, and effort. In contrast, Ultraviolet-Visible (UV-Vis) spectrophotometry is a simple and rapid method that allows for the reliable quantification of drugs [33,34,35]. In this regard, this study aims to investigate the following:

1. Explore the solubility profile of ketamine HCl for IN delivery by manipulating different variables including pH, co-solvent addition, and incorporation of different surfactants with the goal of achieving an effective concentration within a small volume.
2. Develop a validated method for the ketamine HCl analysis using UV-Vis spectrophotometry.

## 2. Material and Methods

### 2.1. Materials

Ketamine HCl was obtained from Supriya Lifescience Ltd., Mumbai, India. PG (CAS No. 57-55-6), poloxamer (PLX) 407 (CAS No. 9003-11-6), sodium dodecyl sulfate (SDS, CAS No. 151-21-3), DMSO (CAS No. 67-68-5), and polyethylene glycol (PEG) 400 (CAS No. 25322-68-3) were purchased from Sigma-Aldrich, St. Louis, MO, USA. PLX 188 (CAS No. 9003-11-6) was purchased from Glentham Life Sciences, Corsham, Wiltshire, UK. Polyvinylpyrrolidone (PVP, CAS No. 9003-39-8) was purchased from Thermo Fisher Scientific, Waltham, MA, USA. Tween 80 (CAS No. 500-019-9), ethanol, lecithin soya (LS, CAS No. 8030-76-0), and methanol were purchased from VWR, Radnor, PA, USA. Other chemicals and co-solvents used were reagent grade and used as directed.

### 2.2. UV-Vis Method Validation

A new method for ketamine HCl analysis using UV-Vis spectrophotometry was validated according to the International Conference on Harmonisation guidelines for the validation of analytical procedures [36,37].

#### 2.2.1. Linearity

Linearity was investigated using ketamine HCl solution in 0.1 M citrate buffer (CB), pH = 3.5, in different concentrations levels (25, 50, 100, 200, 300, 400, and 500 µg/mL). The absorbance of calibration standards was measured using Jenway 7205 UV/Vis 72 Series Diode Array Scanning Spectrophotometer (Jenway, London, UK). UV–vis absorption spectra were recorded using 10-mm quartz cuvettes (Hellma, Müllheim, Germany) of a 1 cm optical path length. The spectrophotometric measurements were performed in triplicate in the range of 200–400 nm. The absorbance was further converted into concentration using the calibration curve of ketamine HCl. The linearity was evaluated using linear regression analysis to evaluate the standard error of intercept, standard error of slope, and correlation coefficient (R^2^).

#### 2.2.2. Selectivity

Selectivity for ketamine HCl was investigated by assessing different blank samples, including different solvents (CB pH 3.5, CB pH 5.5, and phosphate-buffered saline (PBS) pH 7.5) and surfactants (1% SDS, 1% PLX 407, 1% PLX 188, and 1% PEG 400), for potential interference with the quantification of ketamine HCl. In brief, a stock of ketamine HCl (200 µg/mL) was prepared in each condition, followed by analysis in the range 200–400 nm, using a UV/Vis spectrophotometer as described in Section 2.2.1.

#### 2.2.3. Precision

The precision of the method was evaluated using repeatability (intra-day) and intermediate precision (inter-day) at three concentration levels (25, 100, and 250 µg/mL) of ketamine HCl (*n* = 12). Repeatability was evaluated by evaluating different samples at the same concentration and on the same day. The intermediate precision was evaluated by comparing the same sample on different days (1 day, 2 days, and 1 week). Precision was reported as relative standard deviation (RSD) % expressed as follows: (SD/mean) × 100. Accuracy was reported as relative error (RE) % as follows: ∣(Measured Value − True Value)/True Value| × 100. The limit of detection (LOD) was assessed as follows: LOD = 3.3 × σ/S, where σ is the standard deviation of the y-intercept, and S is the slope of the calibration curve. The limit of quantification (LOQ) was calculated as follows: LOQ = 10 × σ/S.

#### 2.2.4. Thermal Stability

The stability of the samples was assessed at three concentration levels (25, 100, and 250 µg/mL) of ketamine HCl on different days (1 day, 2 days, and 1 week). Samples were stored at 2 different conditions (at 4 °C and 25 °C). At each point, the absorbance was measured, and the concentration was calculated using the calibration curve equation. Data were expressed as the mean ± standard deviation (SD) (*n* = 6).

### 2.3. Solubility of Ketamine HCl in Various Solvents

Different solvents (i.e., water, methanol, ethanol, PG, DMSO, and NMP) were used to assess the solubility of ketamine HCl in each solvent. In brief, ketamine HCl was ground and added to a glass vial (500 mg/mL). The contents of the vials were then mixed using a vortex mixer to confirm the presence of any excess amount of ketamine HCl in each vial. Further, vials were attached to a temperature-controlled benchtop orbital shaker for shaking at 240 rpm at 25 °C for 14 h. After, the vial contents were centrifuged for 10 min at 6000× *g*. The obtained supernatant was separated, and the absorbance of each sample was measured in the range 200–400 nm, using a UV/Vis spectrophotometer as described in Section 2.2.1.

### 2.4. Effect of pH on Ketamine HCl Solubility

The solubility of ketamine HCl was assessed in different pH mediums to explore the optimal conditions for ketamine HCl solubilization. A total of 0.1 M of CB and PBS were used as solvents. Ketamine HCl was ground and added in excess to a glass vial (500 mg/mL) of various pH mediums including CB at pH 3.5 and 5.5, and PBS at pH 7.5 and 9.5. Further, vials were shaken at 240 rpm at 25 °C for 14 h. After, the vial content was centrifuged for 10 min at 6000× *g*, the obtained supernatant was separated, and the absorbance of each sample was analyzed using a UV/Vis spectrophotometer, as described in Section 2.2.1.

### 2.5. Effect of Surfactants on Ketamine HCl Solubility

After establishing the optimal pH mediums for ketamine solubilization, the effect of incorporating surfactants on ketamine HCl solubility was assessed. In brief, ketamine HCl was added in excess (500 mg/mL) to various pH mediums including CB at pH 3.5 and 5.5, containing different types of surfactants (e.g., SDS, PEG 400, PVP, Tween 80, LS, and PLX 188). The vial contents were shaken for 14 h at a speed of 240 rpm at 25 °C, followed by sample analysis using a UV/Vis spectrophotometer, as described in Section 2.2.1.

### 2.6. Statistical Analysis

The presented data are expressed as the average of the mean ± SD of three independent replicates (*n* = 3). The statistical significance of the presented data were analyzed using a one-way ANOVA and Dunnett’s test, as required, using GraphPad Prism 10 software (ns: not significant; * *p* < 0.05, ** *p* < 0.01; *** *p* < 0.001, **** *p* < 0.0001 indicate statistical significance).

## 3. Results and Discussion

### 3.1. Method Development for Ketamine Analysis

**Figure 2:**
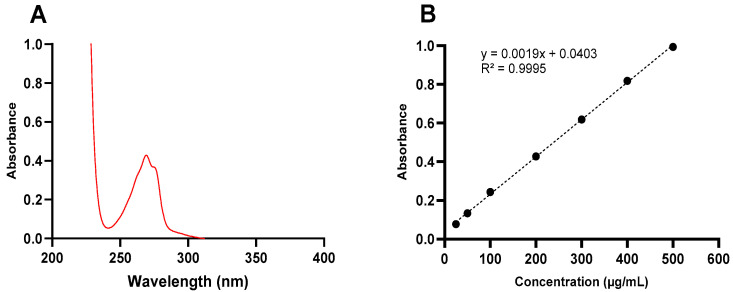
(**A**) UV–Vis absorption spectrum of ketamine HCl (200 µg/mL) in 0.1 M CB, pH = 3.5 between 200 and 400 nm with λ_max_ at 269 nm. (**B**) Calibration curve of ketamine HCl in 0.1 M CB, pH = 3.5 at 269 nm.

The absorption spectra of ketamine HCl in aqueous solution is shown in Figure 2A. The λ_max_ was found to be 269 nm. Because the λ_max_ was found to be similar in all evaluated mediums (water, CB pH 3.5, CB pH 5.5, PBS pH 7.5, and PBS pH 9.5) and was not affected by the pH of the dissolution medium, the same wavelength (269 nm) was used for all further measurements of ketamine HCl concentration. A calibration curve was constructed in the range of seven concentrations (25–500 µg/mL), as shown in Figure 2B. As illustrated in Table 1, the regression equation was y = 0.0019x + 0.0403, with an R^2^ of 0.9995. The LOD refers to the lowest concentration of an analyte that can be reliably detected but not necessarily quantified, while LOQ refers to the lowest concentration of an analyte that can be quantitatively measured with acceptable precision and accuracy, and they were found to be 3.39 and 10.27 µg/mL, respectively. Table 2 illustrates the results obtained for repeatability (intra-day) and intermediate precision (inter-day). In the case of the intra-day analysis, the RSD% results ranged from 1.21% to 4.43%, while the RE% ranged from 2.07 to 2.64%. In the case of the inter-day analysis, the RSD% ranged from 0.52 to 4.32%, while the RE% ranged from 0.56 to 1.08%.

The stability profile of ketamine HCl was assessed under different storage conditions to verify if any spontaneous degradation occurs after the samples are prepared. The results were expressed as the mean ± SD. The obtained data illustrated that the sample solutions were stable for up to 1 week when stored at 4 °C and 25 °C, with a degradation of less than 3% (Table 3). The stability of ketamine HCl when stored at 28 °C in a polypropylene syringe for 48 hours was also reported to be retained for over 95% of its initial concentrations [38]. In addition, the pH measurements remained stable throughout the study, with no signs of physical instability being observed, and thus, ketamine HCl appears to be a stable drug. It is important to note that this method is not intended to serve as a stability-indicating method for monitoring degradation products. Nonetheless, our initial stability testing demonstrated that UV-Vis spectroscopy is adequate for analyzing ketamine HCl. For future long-term stability evaluations, we plan to complement our UV-Vis method with an HPLC-based approach to enhance sensitivity and reliability.

**Figure 3:**
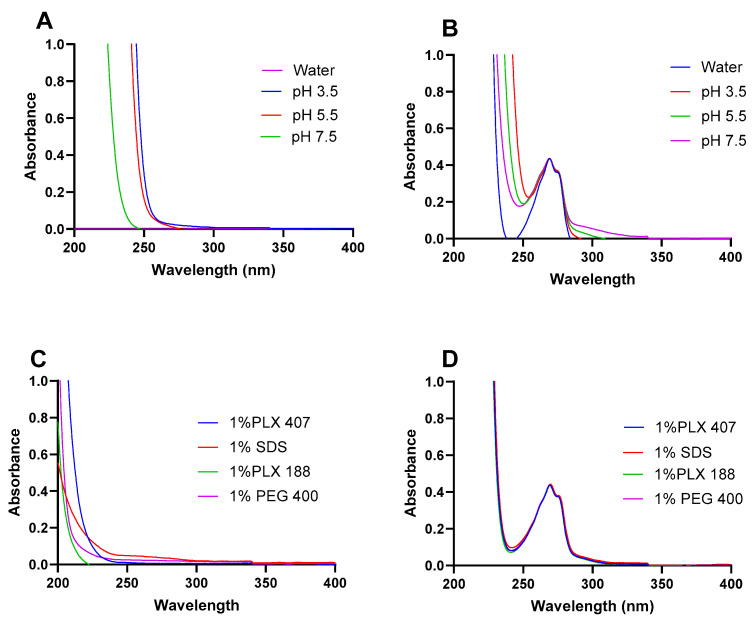
UV–Vis absorption spectrum of ketamine HCl (200 µg/mL) in different solvents without (**A**) and with ketamine HCl (**B**), and in solutions of various surfactants without (**C**) and with ketamine HCl (**D**) in the range of 200–400 nm. Data are expressed as mean ± SD (*n* = 3).

Interference of the solvent or surfactants employed in the solubilization of drugs could lead to inaccurate measurements of the drug’s concentration [39]. In this regard, the interference in the different solvents (water, CB pH 3.5, CB pH 5.5, and PBS pH 7.5) that could be employed during the solubilization of ketamine HCl for IN delivery were evaluated. Interference was assessed by considering whether significant differences were observed at λ_max_ (269 nm). As shown in Figure 3A, no peak was observed in any of the ketamine HCl-free solvents around 269 nm, while the results demonstrate well-defined peaks at the absorbance wavelength of 269 nm in the presence of ketamine HCl (Figure 3B). Moreover, surfactants typically absorb in the ultraviolet region of the spectrum, and thus, could alter the drug quantification using UV-Vis spectrophotometry [40,41]. Therfore, the interference of four surfactants (1% SDS, 1%PLX 407, 1%PLX 188, and 1%PEG 400) was also assessed, and clearly, no interference in the absence of ketamine HCl at λ_max_ (269 nm) was observed (Figure 3C) in comparison to the well-defined peaks at the absorbance wavelength 269 nm, in the presence of ketamine HCl (Figure 3D). The absence of interference stems from the presence of an aromatic moiety in keamine that is absent in all evaluated surfactants in this study, resulting in a defined peak for examination at 269 nm. Similarly, Rapalli et al. also reported that no interference between the employed surfactants and buffers of the release media in the quantification of curcumin was observed [42].

### 3.2. Effect of Different Solvents on Ketamine HCl Solubility

Organic co-solvents were reported to play an important role in the solubilization of IN drugs. For instance, Nayzilam^®^, an example of a midazolam nasal spray that was developed by Proximagen, Ltd. (London, UK) and approved by the Food and Drug Administration (FDA) in 2019, was solubilized using different organic co-solvents, including ethanol and PG [19,43]. Valtoco^TM^ is another formulation of IN diazepam reaching the market in 2020 that was solubilized by adding high amounts of organic cosolvents [43].

**Figure 4:**
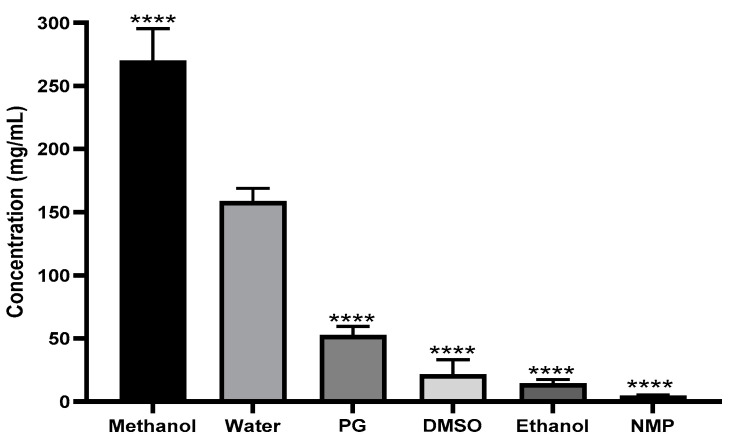
Solubility of ketamine HCl in different co-solvents. Data are expressed as mean ± SD (*n* = 3). Statistical significance was calculated using 1-way ANOVA and Dunnett’s test (**** *p* < 0.0001 indicate statistical significance compared to water).

To identify the possible co-solvent that could be added to water to enhance the solubility of ketamine HCl, we first conducted a study to evaluate the solubility of ketamine HCl in various commonly used organic solvents (methanol, ethanol, PG, DMSO, and NMP) using UV–vis absorption spectrometry with independent calibration curves using the tested solvents. Figure 4 illustrates clearly that the solubility of ketamine was the highest in methanol (269.9 ± 25.4 mg/mL), followed by water (158.5 ± 10.2 mg/mL). The solubility of ketamine in methanol could be explained by many factors, including ketamine’s tendency to form hydrogen bonds with methanol utilizing its ketone functional group (-C=O) and an amine functional group (-NH_2_). Both functional groups could potentially be involved in hydrogen bonding with methanol [44,45,46]. However, the solubility of ketamine HCl in ethanol was more than ten times lower than in methanol, despite ethanol’s similar ability to form hydrogen bonds with ketamine HCl. Figure 4 clearly indicates that the solubility of ketamine HCl in organic solvents is related to solvent polarity. Methanol, with a relative polarity to water of 0.762, exhibits superior solubilization compared to ethanol, which has a relative polarity value of 0.654. It is worth mentioning that methanol was the only organic solvent that exhibited a better solubilization capability compared to water. Unfortunately, the use of methanol as a co-solvent for IN delivery is uncommon and might induce acute toxicity and irritation, and thus, it served only as an experimental tool in the early stages and was excluded from further consideration. Collectively, no common solvent was found to be suitable for significantly boosting the solubility of ketamine HCl in water. Therefore, a completely aqueous system was considered for further investigations.

### 3.3. Effect of pH on Ketamine HCl Solubility

**Figure 5:**
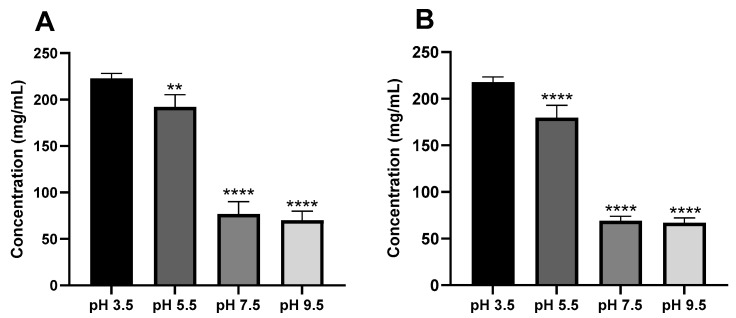
Solubility of ketamine HCl as a function of pH in (**A**) 0.1 M CB and (**B**) 0.1 M PBS. Data are expressed as mean ± SD (*n* = 3). Statistical significance was calculated using 1-way ANOVA and Dunnett’s test (** *p* < 0.01, and **** *p* < 0.0001 indicate statistical significance compared to the pH 3.5 condition).

The solution pH could induce a significant impact on the drug solubility profile, which in turn affects its absorption, biodistribution, and bioavailability in the site of action [47,48]. The nasal mucosa’s pH is in the range of 5.5 to 6.5 [49]. However, nasal formulation pH could exhibit a wider range (3.5–7.5) to consider other factors such as solubilization and stability of therapeutics. In this regard, a wide pH range at 3.5, 5.5, 7.5, and 9.5 was investigated, which is a standard approach in solubility evaluations. To further ensure that the choice of buffer type does not induce any alterations in ketamine solubility, two different buffer systems, including CB and PBS, were tested. This approach was implemented to confirm that the buffers would not interfere with the solubility of ketamine under experimental conditions. Overall, the buffer type did not significantly affect ketamine’s solubility (Figure 5), and a similar solubility profile was obtained in both CB and PBS across all tested pH values. The findings in Figure 5 also illustrate that pH is the primary influencing factor, where the highest solubilization profile of ketamine HCl was observed in acidic environments at pH 3.5 (222.8 ± 5.3 mg/mL at CB and 218.6 ± 5.2 mg/mL at PBS) and this is consistent with ketamine being a basic drug, followed by pH 5.5 (191.9 ± 13.3 mg/mL at CB and 179.4 ± 13.5 mg/mL at PBS). In contrast, ketamine HCl was poorly soluble in basic environments at pH 7.5 (76.7 ± 13.5 mg/mL at CB and 69.0 ± 5.0 mg/mL at PBS) and at pH 9.5 (69.8 ± 10.1 mg/mL at CB and 67.1 ± 5.2 mg/mL at PBS). This could be explained by the fact that ketamine is a weak base, and it possesses a pKa of 7.5, and thus, the protonation of amine (ionization) is promoted at a more acidic pH. However, when pH = pKa, ketamine HCl tends to become 50% protonated and 50% deprotonated, and thus, its solubility decreases, which has been observed at pH 7.5 (Figure 5). While at pH > pKa, the drug will become more deprotonated, and thus, its solubility decreases in the media. In this context, changes in the ionization state of drugs could significantly alter its solubility and PK profiles in different physiological environments. These findings are consistent with the fact that the pH of commercially available ketamine solutions for intravenous injections is maintained at an acidic level (around pH 3.5–5.5) to ensure the maximum stability and solubility of the drug. A systematic evaluation of the effects of buffer composition (type and ionic strength) on solubility was not conducted in this study, but this could be the focus of future studies.

### 3.4. Effect of Surfactants on Ketamine HCl Solubility

**Figure 6:**
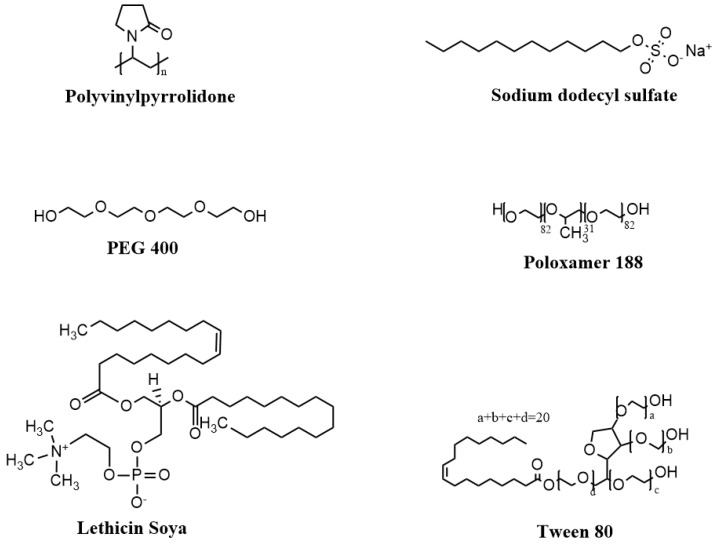
Chemical structures of the used surfactants for ketamine HCl solubilization. Figure was created in ChemDraw.

**Figure 7:**
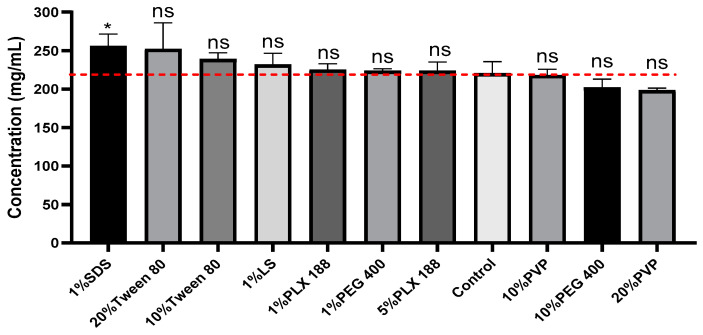
Solubility of ketamine HCl with different surfactants in CB with pH 3.5 (control) with different surfactants. Data are expressed as mean ± SD (*n* = 3). Statistical significance calculated using 1-way ANOVA and Dunnett’s test (ns = not significant, * *p* < 0.05 indicate statistical significance compared to the control).

Surfactants play an important role in solubilizing the poorly soluble drugs, improving their dissolution profile and drug delivery, as well as enhancing their stability profile [26]. The idea behind employing surfactant strategy for improving the solubility of drugs emerged from a reported patent involving the incorporation of surfactants with ketamine in order to reduce the interfacial tension, and thus to reduce the tendency of ketamine HCl to precipitate [50]. To explore their impact on the solubility profile of ketamine HCl, different commonly used pharmaceutical surfactants were investigated under acidic pH environments including SDS, PEG 400, PVP, Tween 80, poloxamer 188, and lecithin Soya (Figure 6). Among the tested surfactants, 1% SDS was the potential surfactant for ketamine HCl solubilization (Figure 7). Under CB at pH 3.5 environment, the solubility of ketamine HCl improved from 222.8 ± 5.3 mg/mL to 256.1 ± 15.4 mg/mL (16% enhancement) in the presence of 1% SDS (Figure 7). Similarly, SDS was previously reported to significantly improve the solubility of various drugs [51,52]. Surprisingly, the solubility of ketamine HCl decreased with the incorporation of 1% PEG 400 and further decreased with 10% PEG 400, as shown in Figure 7. This can be explained by the salting-out effect, where PEG, a hydrophilic polymer, undergoes high solvation in water, reducing water’s capacity to dissolve other molecules, including ketamine HCl. A similar pattern was observed with PVP as a surfactant; the solubility of ketamine HCl decreased more with 20% PVP compared to 10% PVP. This behavior for both PEG and PVP is due to their high solvation in water, leading to the reduced solvation of ketamine HCl. Conversely, the addition of 10% Tween 80 slightly enhanced the solubility of ketamine HCl by approximately 5%, and the addition of 20% Tween 80 enhanced the solubility by around 9%. Unlike PEG 400 and PVP, Tween 80 can form micelles above its critical micelle concentration. This results in a lower salting-out effect and provides a greater solubilization capacity within the hydrophobic compartments of the formed micelles. LS and PLX 188 are two micelle-forming surfactants, and they slightly improved the solubility of ketamine to a lower degree compared to Tween or SDS. Collectively, our results indicate that SDS at 1% w/v is the optimal surfactant for improving the solubility of ketamine HCl, despite only providing marginal enhancement in solubilization.

## 4. Conclusions

This study explored the solubility profile of ketamine HCl for intranasal delivery by manipulating variables such as co-solvent effects, surfactant incorporation, and pH adjustments. The findings revealed a marked improvement in ketamine HCl solubility, particularly through pH optimization and the addition of surfactants like SDS. A preliminary one-week stability assessment showed consistent solubility and pH stability, supporting the preparation of highly concentrated ketamine HCl solutions suitable for IN delivery, which is critical given the limited dose volume per nostril and the high therapeutic doses required.

These outcomes highlight the potential of these solubilization strategies to address the solubility limitations of ketamine HCl, enabling the preparation of highly concentrated ketamine HCl formulations for intranasal delivery, which could improve both the accessibility and efficacy of ketamine-based therapies. Future studies should focus on longer-term stability testing to establish self-life and formulation robustness under various storage conditions. Additionally, applying these approaches to other active pharmaceutical ingredients may broaden intranasal delivery applications, enhancing bioavailability and therapeutic efficacy in clinical settings.

## Figures and Tables

**Table 1:** Results of regression analysis of data for the quantitation of ketamine HCl.

Linear range (µg/mL)	25–500
Regression equation	Y = 0.0019x + 0.0403
Standard error of slope	1.9 × 10^−5^
Standard error of intercept	1.96 × 10^−3^
R^2^ (mean ± SD)	0.9995 ± 0.0074
LOD (µg/mL)	3.39
LOQ (µg/mL)	10.27

**Table 2:** Precision (%, RSD) and accuracy (%, RE) for propofol, ketamine HCl. Data are expressed as mean ± SD (*n* = 12).

Added Concentration (µg/mL)		Intra-Day (n = 12)			Inter-Day (n = 12)		
		Concentration Found (Mean ± SD)	Precision (RSD, %)	Accuracy (RE, %)	Concentration Found (Mean ± SD)	Precision (RSD, %)	Accuracy (RE, %)
	25	25.51 ± 1.13	4.43	2.07	25.16 ± 1.08	4.32	0.66
	100	102.35 ± 2.36	2.31	2.35	100.56 ± 1.83	1.82	0.56
	250	256.61 ± 3.12	1.21	2.64	252.71 ± 1.33	0.52	1.08

**Table 3:** Stability results of ketamine HCl at room temperature (25 °C) and in the refrigerator (4 °C). Data are expressed as mean ± SD (*n* = 6).

		Concentration (µg/mL) (Mean ± SD)		
		25	100	250
Room temperature (25 °C)	Day 1	24.26 ± 3.71	100.64 ± 0.92	255.91 ± 0.30
	Day 2	24.46 ± 1.48	99.77 ± 0.97	253.10 ± 5.26
	1 week	25.82 ± 0.90	103.89 ± 0.28	250.12 ± 0.30
Refrigerator (4 °C)	Day 1	24.15 ± 1.15	100.29 ± 0.79	255.38 ± 3.65
	Day 2	25.12 ± 0.90	97.00 ± 1.26	254.42 ± 3.58
	1 week	25.21 ± 1.33	100.82 ± 2.57	254.50 ± 3.30

## Data Availability

The data supporting this article have been included in this paper.
